# The sympathetic nervous system is controlled by transient receptor potential vanilloid 1 in the regulation of body temperature

**DOI:** 10.1096/fj.15-272526

**Published:** 2015-07-01

**Authors:** Khadija M. Alawi, Aisah A. Aubdool, Lihuan Liang, Elena Wilde, Abhinav Vepa, Maria-Paraskevi Psefteli, Susan D. Brain, Julie E. Keeble

**Affiliations:** *Institute of Pharmaceutical Science and ^†^British Heart Foundation Cardiovascular Centre of Excellence and Centre of Integrative Biomedicine, Cardiovascular Division, King’s College London, London, United Kingdom

**Keywords:** TRPV1, thermogenesis, brown adipose tissue

## Abstract

Transient receptor potential vanilloid 1 (TRPV1) is involved in sensory nerve nociceptive signaling. Recently, it has been discovered that TRPV1 receptors also regulate basal body temperature in multiple species from mice to humans. In the present study, we investigated whether TRPV1 modulates basal sympathetic nervous system (SNS) activity. C57BL6/J wild-type (WT) mice and TRPV1 knockout (KO) mice were implanted with radiotelemetry probes for measurement of core body temperature. AMG9810 (50 mg/kg) or vehicle (2% DMSO/5% Tween 80/10 ml/kg saline) was injected intraperitoneally. Adrenoceptor antagonists or vehicle (5 ml/kg saline) was injected subcutaneously. In WT mice, the TRPV1 antagonist, AMG9810, caused significant hyperthermia, associated with increased noradrenaline concentrations in brown adipose tissue. The hyperthermia was significantly attenuated by the β-adrenoceptor antagonist propranolol, the mixed α-/β-adrenoceptor antagonist labetalol, and the α_1_-adrenoceptor antagonist prazosin. TRPV1 KO mice have a normal basal body temperature, indicative of developmental compensation. d-Amphetamine (potent sympathomimetic) caused hyperthermia in WT mice, which was reduced in TRPV1 KO mice, suggesting a decreased sympathetic drive in KOs. This study provides new evidence that TRPV1 controls thermoregulation upstream of the SNS, providing a potential therapeutic target for sympathetic hyperactivity thermoregulatory disorders.—Alawi, K. M., Aubdool, A. A., Liang, L., Wilde, E., Vepa, A., Psefteli, M.-P., Brain, S. D., Keeble, J. E. The sympathetic nervous system is controlled by transient receptor potential vanilloid 1 in the regulation of body temperature.

Transient receptor potential vanilloid 1 (TRPV1) is a nonselective cation channel, predominantly expressed on perivascular sensory neurons (1). It is widely considered as an integrator of noxious stimuli, including capsaicin, noxious heat, and acid conditions, among others. TRPV1 channels presented a novel target for pharmacologic manipulation for inflammatory pain conditions (1, 2). More recently, it has been found that TRPV1 plays a fundamental role in basal body temperature regulation in a variety of species from mice to humans (3, 4). Acute inhibition of TRPV1 causes hyperthermia, reaching a body temperature of ∼40°C in 1 human subject. However, the precise mechanisms underlying this hyperthermia are not fully understood.

Mammalian body temperature is regulated constantly by a fine balance between heat loss and generation (5); thermoregulatory homeostasis is achieved *via* behavioral and physiologic effector responses. This is principally achieved by the autonomic nervous system, through intricate circuits involving peripheral thermosensors and the CNS to mediate effector mechanisms in response to changes in the ambient temperature (6). Cold exposure stimulates the sympathetic nervous system (SNS), where heat-gain mechanisms involving thermogenesis in brown adipose tissue (BAT) are activated (6). Additional effector responses include cutaneous constriction, thereby combining heat production *via* BAT-derived thermogenesis, and retention of heat *via* cutaneous constriction (6). However, BAT-mediated thermogenesis is the most potent thermogenic effector mechanism and is exclusively mediated by uncoupling protein (UCP)1, downstream of β-adrenoceptor activation (7). This process induces and activates mitochondrial UCP1, which uncouples oxidative phosphorylation from ATP production, releasing chemical energy as heat (8). Although BAT has been previously considered to be present in newborn humans, in addition to a fundamental role in rodents, hibernating mammals (9), BAT has now been shown to be functionally expressed in adults (10, 11). Additionally, humans with metabolically active BAT depots respond to a β3-adrenoceptor agonist, which stimulated BAT metabolic activity and enhanced global metabolism (12).

Hyperthermia, induced by TRPV1 inhibition, has been shown to result in increased oxygen consumption, coupled with tail skin vasoconstriction in rodents, which are characteristic thermoeffectors downstream of autonomic activity (13). This suggests that the hyperthermia associated with TRPV1 inhibition mimics sympathetically mediated thermogenesis.

As inhibition of TRPV1 results in hyperthermia, it was expected that TRPV1 knockout (KO) mice would exhibit altered thermoregulatory pathways. However, TRPV1 KO mice do not exhibit gross differences in their core body temperatures under neutral ambient conditions (3). A similar phenomenon is observed in wild-type (WT) mice that are chronically treated with TRPV1 antagonist (14), suggesting that sympathetic drive has been reduced as a compensatory mechanism to normalize body temperature in these animals.

In the present study, we have used a pharmacological approach, using the TRPV1 antagonist, AMG9810, to investigate the role of TRPV1 in basal body thermoregulation. We subsequently sought to explore the thermoregulatory profile of TRPV1 KO mice. Based on all of the current evidence discussed above, we tested the hypotheses that TRPV1 inhibition results in hyperthermia due to disinhibition of the SNS and that TRPV1 KO mice exhibit a suppressed sympathetic drive to maintain thermoregulatory homeostasis.

## MATERIALS AND METHODS

### Ethics statement

All experiments were conducted in accordance with the United Kingdom Home Office Animals (Scientific Procedures) Act 1986 and Amendment Regulations 2012. They were also approved by the King’s College London Animal Welfare and Ethical Review Body.

### Animals

Male mice (8–15 wk of age) were used for all experiments. Animals were housed in temperature- (22 ± 2°C) and humidity-controlled (50 ± 10%) colony rooms maintained under filtered positive pressure ventilation on a 12-h light-dark cycle beginning at 7:00 am Greenwich mean time with free access to water and food. Male, age-matched C57BL6/129SvJ WT and TRPV1 homozygous KO mice (with >7 generations of backcrosses) were used at 8 wk of age. TRPV1 KO mice were generated by replacing the exon, which encodes part of the fifth and entire sixth transmembrane domain (15). The genotype of each animal was established by PCR as previously described (16, 17). All recovery procedures were performed under isoflurane anesthesia (2% volume isoflurane and 2% volume O_2_) for induction and maintenance. Blood samples were collected *via* the left ventricle of the heart by cardiac puncture to obtain plasma; animals were killed by cervical dislocation under anesthesia. Plasma was separated by centrifugation (2000 *g* for 20 min).

### Radiotelemetry surgical implantation

Male WT and TRPV1 KO were used for all remote radiotelemetry studies, as previously described (18). Buprenorphine analgesia was administered intramuscularly perioperatively (10 µg/kg; Vetersergic; Sogeval, Sheriff Hutton, United Kingdom). Mice were anesthetized (2–3% volume isoflurane carried in 2–3% volume O_2_), the abdomen was shaved and scrubbed using surgical iodine, and the mice were placed on a homeothermic heating mat. A small ventral midline abdominal incision (<1 cm) was made, and the abdominal muscle wall was exposed. A ventral incision was made on the abdominal wall and was irrigated with sterile saline (0.9% saline; sodium chloride, pyrogen free) to facilitate the insertion of the radiotelemetry transmitter [TA10TA-F10; Data Science International (DSI), St. Paul, MN, USA]. Following implantation, the abdominal wall and the skin incision were sutured separately using absorbable sutures (Vicryl 4.0; Ethicon, Johnson & Johnson, New Brunswick, NJ, USA). The mice were monitored until ambulatory in an incubator maintained at 26°C and were then individually housed with food and water *ad libitum*. Animals were allowed at least a 7 d postsurgical recovery period. Cages containing the telemetered animals were placed on the receiver plates (RPC-1; DSI); radio signals from the implanted transmitters were monitored *via* a fully automated data acquisition system (Dataquest A.R.T., version 4.1; DSI). Core body temperature and locomotor activity were monitored for 10 min intervals, for durations of 120 s. Core body temperature and activity were monitored daily; baseline measurements were recorded for at least 60 min pretreatment. Data were collected and analyzed in Microsoft Excel (Microsoft, Redmond, WA, USA) and GraphPad Prism 5 (GraphPad Software, La Jolla, CA, USA).

### Acute immobilization studies

WT mice were anesthetized with ketamine (75 mg/kg) and medetomidine (1 mg/kg). All mice were placed on a heat blanket, the temperature of which was determined by homeothermically coupling it to control, untreated mice. A temperature microchip transmitter (LifeChip; Destron Fearing, Eagan, MN, USA) was inserted intraperitoneally following a small incision to the skin and abdominal layer; the incisions were sutured as described previously. Body temperature was monitored every 15 min, and following a baseline period, AMG9810 (50 mg/kg; 5 ml/kg, i.p.) or vehicle (2% DMSO, 5% Tween 80 in saline) was administered. Body temperature was monitored for 120 min thereafter.

### Drugs

All agents were from Sigma-Aldrich (Poole, United Kingdom), unless stated otherwise. Doses of drugs administered were obtained from literature or optimized from published doses. Adrenoceptor antagonists or control was administered subcutaneously 30 min prior to administration of the TRPV1 antagonist AMG9810 (E)-3-(4-*t*-butylphenyl)-*N*-(2,3-dihydrobenzo[b][1,4] dioxin-6-yl)acrylamide) [50 mg/kg; 10 ml/kg, i.p. (19)] or vehicle (2% DMSO, 5% Tween 80 in saline; 10 ml/kg, i.p.). Propranolol hydrochloride [5 mg/kg (20)], labetalol hydrochloride [30 mg/kg (21, 22)], prazosin hydrochloride [0.1 mg/kg (23)], ICI 118,551 hydrochloride [10 mg/kg (24)], and metoprolol hydrochloride [20 mg/kg (25)] were dissolved in saline. The selective β_3_-adrenoceptor antagonist SR59230A (2S)-1-(2-ethylphenoxy)-3-[[(1S)-1,2,3,4-tetrahydronaphthalen-1-yl]amino]propan-2-ol [2.5 mg/kg (26)] was dissolved in 2% DMSO and suspended in saline. In separate experiments, d-amphetamine hemisulfate was suspended in saline and administered subcutaneously at a dose of 10 mg/kg (27). Because circadian factors are known to influence core body temperature, all dosing was initiated between 12:00 pm and 1:00 pm. Treatments were randomized, and the experimenter was blinded to the genotype of animals at the time of experiments.

### Calculation of the hyperthermic index

For each animal, the area under the curve (AUC) for temperature was calculated using GraphPad Prism 5 software. Briefly, data for 60 min pretreatment were used as a mean baseline reading to calculate individual AUC values utilizing the analysis function provided by the software. The time points analyzed corresponded to each hour posttreatment, and the results from individual mice were grouped, analyzed, and presented as a hyperthermic index (°C × min) or activity index (activity counts × min), for studies using d-amphetamine.

### Quantification of noradrenaline concentrations

Noradrenaline (NA) concentrations were measured using a commercially available NA ELISA kit (IBL International, Hamburg, Germany), as previously described (28). Tissue was collected in a sterile environment and snap frozen at −80°C until processing. Tissue was homogenized in lysis buffer [RIPA; 1% NP-40, 0.1% sodium dodecyl sulfate, 50 mM Tris-HCl, 150 mM NaCl, 0.5% sodium deoxycholate, and 1 mM EDTA (pH 7.4)] containing protease inhibitors (1 tablet/50 ml; Roche Diagnostics, Burgess Hill, United Kingdom), and lysates were obtained by centrifuging at 2600 *g* for 10 min at 4°C. According to the manufacturer’s instructions, 20 μl tissue lysates, standards, and controls was added to extraction plates. After extraction, bound NA (25 µl) was eluted using release buffer and transferred to a 96 well ELISA plate. A total of 50 µl NA antiserum was incubated with all samples at room temperature for 120 min on an orbital shaker; the plates were thoroughly washed with diluted washing buffer, and 100 µl enzyme conjugate was added into each well and incubated for 60 min at room temperature. Following several washes, 200 µl p-nitrophenyl phosphate (pNPP) substrate solution was incubated at room temperature for 40 min, and the reactions were stopped by addition of 50 µl pNPP stop solution per well. The optical densities of each well were measured at 405 nm, and a standard curve was plotted using a range of known NA concentrations provided in the kit (0, 5, 15, 50, 150, and 500 ng/ml). Positive and negative controls were included to determine accuracy. The limit of sensitivity was 20 pg/ml, and the linearity limit was 8.0 ng/ml. Cross-reactivity to other catecholamines or metabolites was manufacturer tested as <0.02%. Protein concentrations of each sample were determined using the Bradford dye-binding method (Bio-Rad, Hemel Hempstead, United Kingdom), and NA concentrations of each sample were normalized to milligrams of protein and expressed as NA (nanograms per milligram of tissue protein) (29).

### Measurement of plasma cytokines

At 2 h post-AMG9810, blood samples were collected to obtain plasma under isoflurane anesthesia (2–3% volume isoflurane carried in 2–3% volume O_2_). Mouse TNF-α, IL-6, and IL-1β ELISA kits (Invitrogen, Life Technologies, Paisley, United Kingdom) were used to quantify plasma concentrations according to the manufacturer’s instructions. Briefly, 50–100 µl standard, controls, and plasma samples was loaded into the wells. Subsequently, 50 µl biotinylated secondary antibody was added, and plates were incubated at 37°C for 120–180 min. Plates were washed, and 100 µl streptavidin-horseradish peroxidase (HRP) was added to each well and incubated at room temperature for a further 30 min. Stabilized chromogen (100 µl) was added to each well, and the plates were incubated in the dark for 30 min at room temperature. The reaction was stopped with the addition of 100 µl stop solution. The optical densities of each well were measured at a wavelength of 450 nm, and a standard curve was generated to calculate the sample cytokine concentrations. The limit of sensitivity was <3 pg/ml, and the linearity limit was 6 pg/ml. Cross-reactivity to other cytokines was manufacturer tested as <0.01%.

### Quantitative PCR

RNA was extracted from BAT and *gastrocnemius* (GM) samples using the Qiagen RNeasy Microarray kit (Qiagen, Crawley, United Kingdom) following the manufacturer’s instructions. RNA was quantified by optical density measurements on a Nano-Drop 8000 Spectrophotometer (Thermo Scientific, Loughborough, United Kingdom). Total RNA (0.5–1 μg) was reverse transcribed using the high-capacity RNA-to-cDNA kit supplemented with RNAse inhibitor according to the manufacturer’s instructions (Applied Biosystems, Life Technologies) in a 20 μl reaction volume. Quantitative PCR (qPCR) was performed with the SensiMix SYBR No-ROX Kit (Bioline, London, United Kingdom) with Hot-Start Taq polymerase on a Corbett Rotorgene using predesigned primers from Sigma-Aldrich (**Table 1**). Samples were heated to 95°C for 10 min (initial denaturation), followed by 45 cycles of 10 s at 95°C, 15 s at 57°C, and 5 s at 72°C; melt was 68–90°C with fluorescence detection after each cycle. Samples were subjected to melting curve analysis to confirm amplification specificity. Samples were normalized to hypoxanthine phosphoribosyltransferase (HPRT) and expressed as relative fold change using the ^ΔΔ^*C_t_* method of relative quantification as described by Livak and Schmittgen (30).

**TABLE 1.** qPCR primer information

Target	Primer sequence	Accession number	Product length (bp)
Dio2	F: TCCTAGATGCCTACAAACAGGTTA	NM_010050	148
	R: GCACTGGCAAAGTCAAGAAGG		
HPRT	F: TCCTCCTCAGACCGCTTTT	NM_013556.2	90
	R: CCTGGTTCATCATCGCTAATC		
PGC1-α	F: CCACAGAAAACAGGAACAGCAG	NM_008904.2	142
	R: CCCTTGGGGTCATTTGGTGA		
PPAR-γ	F: GCCTATGAGCACTTCACAAGAAAT	NM_001127330	173
	R: TGCTGGAGAAATCAACTGTGGT		
UCP1	F: GAGGTGTGGCAGTGTTCATT	NM_009463	112
	R: TAAGCATTGTAGGTCCCCGTGT		
UCP2	F: AGATGTGGTAAAGGTCCGCTTC	NM_011671.4	90
	R: GCAATGGTCTTGGTAGGCTTC		
UCP3	F: TTCCCCTGTCACTTTGTCTCTG	NM_009464.3	85
	R: ATCGGGTCTTTACCACATCCAC		

F, forward; R, reverse.

### Immunoblotting

Interscapular BAT and GM from male TRPV1 WT and KO mice and WT mice administered with AMG9810 or vehicle were immediately dissected and snap frozen in liquid nitrogen for mitochondrial protein extraction using a Mitochondria Isolation Kit (Thermo Scientific) according to the manufacturer’s instructions. Equal amounts of protein (15–30 μg) were separated by SDS-PAGE under reducing conditions in 10% polyacrylamide gels. The proteins were transferred to PVDF membranes (EMD Millipore, Feltham, United Kingdom), blocked in 5% nonfat dried milk (1 h at room temperature), and incubated with primary antibody for UCP1 and UCP3 (1:1000 ab23841 and ab3477; Abcam, Cambridge, United Kingdom) at 4°C for 12 h. The voltage-dependent anion-selective channel (VDAC) was used as a loading control (1:1000 AB10527; EMD Millipore). The membranes were exposed under ECL (Luminata Classico Western HRP substrate; EMD Millipore) in a G-BOX Gel Documentation System (Syngene, Cambridge, United Kingdom) using the manufacturer’s software (Syngene 2D gel imaging software; Syngene). Samples were quantified densitometrically using ImageJ (National Institutes of Health, Bethesda, MD, USA).

### Histologic assessment of BAT

Intrascapular BAT pads were fixed in 4% paraformaldehyde in PBS, embedded in paraffin, and 10 μm sections were assessed for general morphology using hematoxylin and eosin staining, as described by Shimizu *et al.* (31). Large lipid droplets were defined as lipids >25 μm, measured with ImageJ, and counted per field at ×200 magnification. A minimum of 10 fields was randomly selected from each sample (*n* = 4–6 mice per experimental group), and results are presented as lipid droplets (micrometers per field).

### Analysis

Results are presented as means ± sem. Statistical differences between samples were determined by either unpaired Student’s *t* test or 2-way ANOVA and Bonferroni *post hoc* test using the GraphPad Prism software [version 5.02 for Windows (Microsoft)]. *P* < 0.05 was considered to represent a significant difference.

## RESULTS

### AMG9810-induced hyperthermia is dependent on spontaneous activity

**Figure 1.**
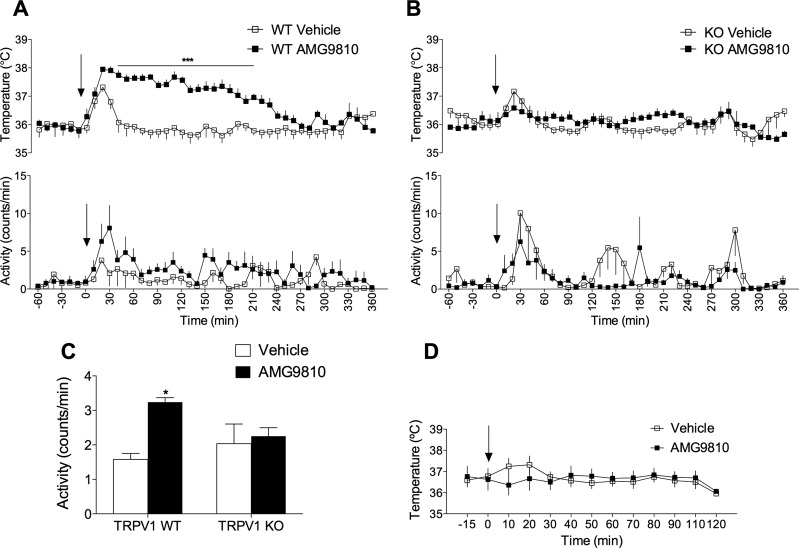
AMG9810-induced hyperthermia is dependent on spontaneous activity. *A*) Core body temperature (top) and activity (bottom) recordings over 360 min following AMG9810 (50 mg/kg, i.p.) or vehicle (2% DMSO, 5% Tween 80 in saline) in TRPV1 WT mice. *B*) Temperature (top panel) and activity (bottom panel) profile of AMG9810 in TRPV1 KO mice (*n* = 7–8 per group). Arrows in (*A*) and (*B*) denote treatments. *C*) A 3 h average activity posttreatment (*n* = 7–8 per group). *D*) Administration of AMG9810 or vehicle to anesthetized WT mice (*n* = 5–6 per group). Results are means ± sem. *P < 0.05 and ***P < 0.001 *vs.* vehicle control using 2-way ANOVA and Bonferroni’s.

The initial aim of the present study was to characterize the hyperthermia induced by the polymodal antagonist, AMG9810, and confirm that the effect on body temperature was on target (**Fig. 1*A***). Indeed, TRPV1 KO mice were immune to the AMG9810-induced hyperthermia (Fig. 1*B*). Additionally, analysis of activity levels in WT mice showed a sustained increase in spontaneous activity in AMG9810-treated mice (Fig. 1*A*, *C*), whereas vehicle-treated mice exhibited a transient increase in activity associated with the injection artifact. This increase in activity (hyperkinesis) is in agreement with previous findings by other authors (18). Thus, to establish the contribution of spontaneous activity to the hyperthermia, AMG9810 was administered to anesthetized mice (Fig. 1*D*). AMG9810 failed to elicit the typical acute hyperthermia in these mice, demonstrating a positive relationship between spontaneous activity and hyperthermia.

The clinical distinction between fever and hyperthermia is the disruption of the thermoregulatory set point in the former, typically cytokine driven (32), whereas hyperthermia involves deficits in thermoregulatory mechanisms such as heat dissipation with no implication of central mechanisms. Plasma cytokine concentrations were measured 2 h posttreatment with AMG9810, and no significant increase in IL-6, TNF-α, or IL-1β concentrations was observed (Supplemental Fig. 1), confirming the lack of fever in these animals.

### AMG9810-induced hyperthermia is sympathetically driven

**Figure 2.**
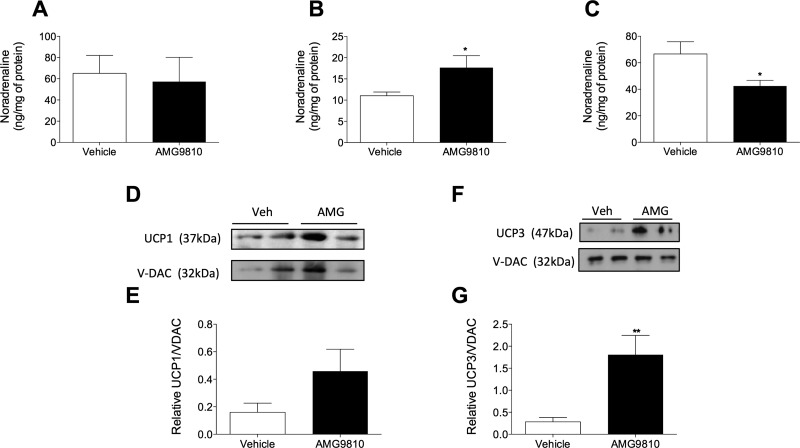
Involvement of the SNS in TRPV1 antagonist-induced hyperthermia. NA concentrations measured in brain (*A*), BAT (*B*), and skin (*C*) samples of WT mice 1 h posttreatment with AMG9810 (50 mg/kg, i.p.) or vehicle (2% DMSO, 5% Tween 80 in saline) (*n* = 5). *D*) Immunoblot of UCP1 and loading control, VDAC, in BAT mitochondrial-extracted protein of AMG9810- or vehicle (Veh)-treated mice (1 h). *E*) Densitometric analysis of the relative expression of UCP1/VDAC (*n* = 3–4). *F*) Immunoblot of UCP3 and loading control, VDAC, in skeletal muscle (GM) mitochondrial-extracted protein of AMG9810- or vehicle-treated mice (1 h). *G*) Densitometric analysis of the relative expression of UCP3/VDAC (*n* = 4). Results are means ± sem. *P < 0.05 and **P < 0.01 *vs.* vehicle control using 2-tailed Student’s *t* test.

As it was established that AMG9810 induces a pharmacologic hyperthermia, as opposed to cytokine-induced fever, we hypothesized that TRPV1 antagonist-induced hyperthermia involves activation of the SNS. To test this hypothesis, NA concentrations were measured in WT mice at the peak onset of the hyperthermia, 1 h posttreatment with AMG9810 or vehicle. The NA content of brain samples from AMG9810 compared with vehicle-treated mice did not differ (**Fig. 2*A***), suggesting a lack of central NA activity in this hyperthermia. Additionally, kidney NA concentrations were unaltered (48.52 ± 6.9 *vs.* 52.05 ± 18.90 ng/ml), suggesting no involvement of renal adrenergic pathways. In contrast, AMG9810 caused a significant increase in the NA content of BAT (Fig. 2*B*), which suggests an increase in sympathetic activity and BAT-mediated thermogenesis. Interestingly, a significant decrease in NA content in skin samples of AMG9810-treated animals was observed (Fig. 2*C*), compared with vehicle-treated controls. A modest increase in plasma NA concentrations in AMG9810-treated mice compared to vehicle-treated mice was determined (33.39 ± 12.95 *vs.* 6.162 ± 0.6045 ng/ml; *P* = 0.0805; *n* = 5), suggesting an increase in NA outflow to the periphery.

The transcriptional and protein levels of thermogenic mediators under sympathetic control were determined in BAT additionally, in skeletal muscle. Assessment of UCP1 in BAT demonstrated an induction in AMG9810-treated mice (Fig. 2*D*, *E*); however, this did not reach significance. Although UCP1 is the most potent thermogenic protein in mammals, a skeletal muscle-abundant homolog, UCP3, is additionally involved in nonshivering thermogenesis (33). A significant induction in UCP3 levels was observed in WT mice administered with AMG9810 in comparison with control (Fig. 2*F*, *G*).

Assessment of relative mRNA expression in BAT and GM samples of WT mice administered with AMG9810 or vehicle (**Table 2**) demonstrated no alteration in UCP1 expression. However, a significant induction of the transcription coactivator, peroxisome proliferator-activated receptor γ coactivator 1-α (PGC1-α), in BAT samples from AMG9810-treated mice was observed. It is known that the expression of UCP1 is highly fluidic and is driven by several transcriptional components, including PGC1-α (34). PGC1-α is known to regulate oxidative metabolism by increasing mitochondrial gene expression and function (35). Peroxisome proliferator-activated receptor-γ (PPAR-γ), a regulator of UCP1 expression (34), was unaffected by AMG9810. In contrast, a significant induction of type II iodothyronine deiodinase (Dio2) was observed, implicating the hypothalamic-pituitary-thyroid (HPT) axis in AMG9810-induced hyperthermia.

**TABLE 2.** Relative mRNA expression of thermogenic mediators in AMG9810 or vehicle-pretreated TRPV1 WT and KO mice

Target	Tissue	WT vehicle	WT AMG9810	KO vehicle	KO AMG9810
UCP1	BAT	1.03 ± 0.18	0.50 ± 0.11	0.26 ± 0.06	0.12 ± 0.11
	GM	1.09 ± 0.28	0.94 ± 0.21	0.65 ± 0.10	0.92 ± 0.18
UCP2	BAT	1.00 ± 0.03	1.31 ± 0.42	0.68 ± 0.12	0.11 ± 0.02
	GM	0.91 ± 0.69	0.62 ± 0.18	0.49 ± 0.21	0.70 ± 0.21
UCP3	GM	1.03 ± 0.17	0.63 ± 0.07	0.94 ± 0.04	0.44 ± 0.03
	GM	0.63 ± 0.01	1.11 ± 0.36	0.48 ± 0.23	0.70 ± 0.12
PGC1-α	BAT	0.69 ± 0.16	2.07 ± 0.48**	0.97 ± 0.22	0.61 ± 0.10^##^
	GM	1.02 ± 0.16	0.74 ± 0.01	0.79 ± 0.39	1.13 ± 0.26
PPAR-γ	BAT	1.01 ± 0.10	1.47 ± 0.30	1.37 ± 0.19	0.86 ± 0.11
	GM	1.57 ± 0.80	0.96 ± 0.29	0.72 ± 0.26	0.56 ± 0.31
Dio2	BAT	1.21 ± 0.49	3.03 ± 0.30***	2.14 ± 0.48	1.25 ± 0.28^###^
	GM	1.16 ± 0.36	1.41 ± 0.27	0.72 ± 0.07	1.70 ± 0.24

Results are means ± sem. *n* = 3–5, normalized to HPRT, relative to WT vehicle mice. **P < 0.01 and ***P < 0.001 *vs.* control; ^##^*P* < 0.01 and ^###^*P* < 0.001 WT AMG910 as determined by 2-tailed Student’s *t* test.

### Adrenoceptors contribute to AMG9810-induced hyperthermia

**Figure 3.**
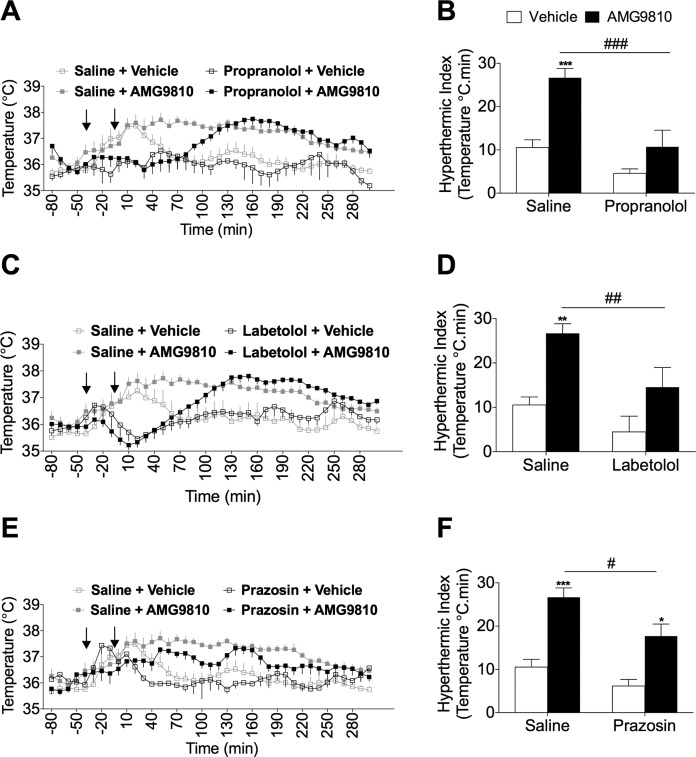
Contribution of adrenoceptors to TRPV1 antagonist-induced hyperthermia. *A*) Core body temperature recordings over 300 min following pretreatment with propranolol (5 mg/kg, s.c.; *n* = 7) or control (saline, 5 ml/kg, s.c.; *n* = 13). *B*) The hyperthermic index was analyzed as area under the temperature curve during the 0 to 180 min period for saline and propranolol-pretreated mice. *C*) Core body temperature recordings over 300 min following pretreatment with labetalol (30 mg/kg, s.c.; *n* = 8) or control (saline, 5 ml/kg, s.c.; *n* = 13). *D*) Respective hyperthermic index calculated for (*C*). *E*) Core body temperature recordings over 300 min following pretreatment with prazosin (0.1 mg/kg, s.c.; *n* = 7) or control (saline, 5 ml/kg, s.c.; *n* = 13) followed by AMG9810 (50 mg/kg, i.p.) or vehicle (2% DMSO, 5% Tween 80 in saline) in WT mice. Arrows in (*A*), (*C*), and (*E*) denote treatments. *F*) The respective hyperthermic index calculated for (*E*). Results are means ± sem. *P < 0.05, **P < 0.01, and ***P < 0.001 *vs.* vehicle control; ^#^*P* < 0.05, ^##^*P* < 0.01, and ^###^*P* < 0.001 *vs.* AMG9810-treated mice using 2-way ANOVA and Bonferroni’s *post hoc* test.

To determine the contribution of postjunctional adrenoceptors to the hypothermia induced by AMG9810, we pretreated mice with adrenoceptor antagonists prior to challenging them with AMG9810. The acute temperature increase following AMG9810 administration demonstrated that the hyperthermia was sustained from 60 to 200 min posttreatment (**Fig. 3*A***). The nonselective β-adrenoceptor antagonist, propranolol, delayed the onset of AMG9810-induced hyperthermia (120 min), compared to saline-pretreated mice (Fig. 3*A*). Analysis of the AUC for the hyperthermic period (defined as the hyperthermia index) demonstrated a significant difference between control-pretreated and propranolol-pretreated mice (Fig. 3*B*). Similarly, the mixed α-/β-adrenoceptor antagonist, labetalol (36), resulted in a marked delay in the onset of the AMG9810-induced hyperthermia (Fig. 3*C*) and a significant reduction in the hyperthermic index (Fig. 3*D*). To investigate the involvement of distinct subtypes of α- and β-adrenoceptors, selective antagonists were consequently used. The effect of selective α_1_-adrenoceptor blockade was assessed, showing that prazosin decreased both the magnitude of the hyperthermia (Fig. 3*E*) and the hyperthermia index (Fig. 3*F*). These results are in agreement with the role of α_1_-adrenoceptors in mediating cutaneous vasoconstriction downstream of NA (37), and thus, treatment with prazosin may have facilitated enhanced heat loss in these mice. Furthermore, prazosin has previously been shown to potently inhibit methylenedioxymethamphetamine (MDMA)-mediated hyperthermia in rodents (26).

**Figure 4.**
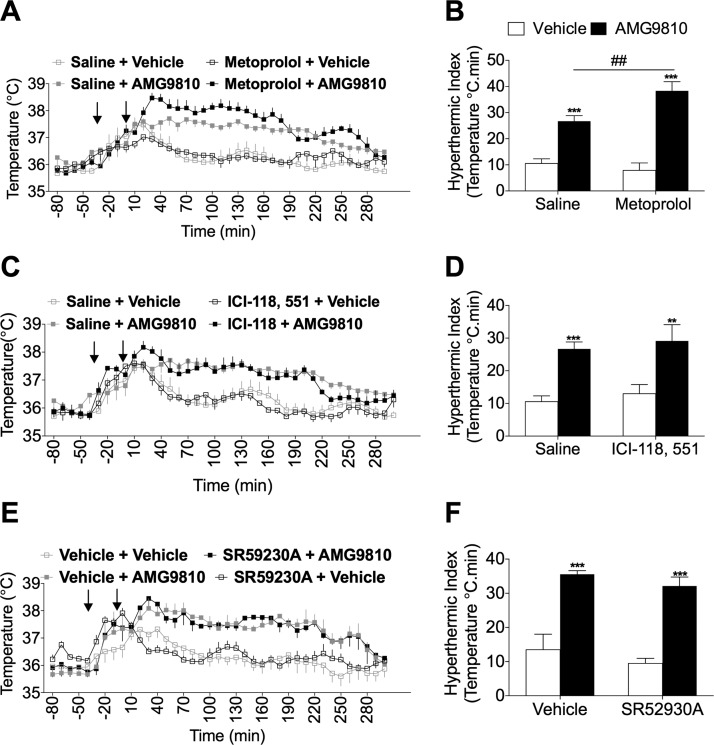
Investigating a role for β-adrenoceptors in TRPV1 antagonist-induced hyperthermia. *A*) Core body temperature recordings over 300 min following pretreatment with metoprolol (20 mg/kg, s.c.; *n* = 6) or control (saline, 5 ml/kg, s.c.; *n* = 13). *B*) The hyperthermic index was analyzed as area under the temperature curve during the 0 to 180 min period for saline and metoprolol-pretreated mice. *C*) Core body temperature recordings over 300 min following pretreatment with ICI-118,551 (10 mg/kg, s.c.; *n* = 6) or control (saline, 5 ml/kg, s.c.; *n* = 13). *D*) Respective hyperthermic index calculated for (*C*). *E*) Core body temperature recordings over 300 min following pretreatment with SR59230A (2.5 mg/kg, s.c.; *n* = 3) or control (2% DMSO in saline, 5 ml/kg, s.c.; *n* = 4). Arrows in (*A*), (*C*), and (*E*) denote treatments. Results are means ± sem. **P < 0.01, and ***P < 0.001 *vs.* vehicle control; ^##^*P* < 0.01 *vs.* AMG9810-treated mice using 2-way ANOVA and Bonferroni’s *post hoc* test.

Selective blockade of β_1_-adrenoceptors with metoprolol resulted in a potentiation of AMG9810-induced hyperthermia (**Fig. 4*A***), where the hyperthermic index was significantly larger in the metoprolol-pretreated group, compared to saline-pretreated groups (Fig. 4*B*). Additionally, selective blockade of β_2_-adrenoceptors with ICI-118,551 did not result in any effect on the hyperthermia (Fig. 4*C*, *D*). Atypical β_3_-adrenoceptors were hypothesized to be involved because they were shown to be involved in the hyperthermia mediated by 3,4-MDMA (26). However, no difference was observed in AMG9810-induced hyperthermia following pretreatment with the β_3_-antagonist, SR59230A, or vehicle (Fig. 4*E*, *F*).

### Thermoregulatory phenotype of TRPV1 KO mice at baseline

**Figure 5.**
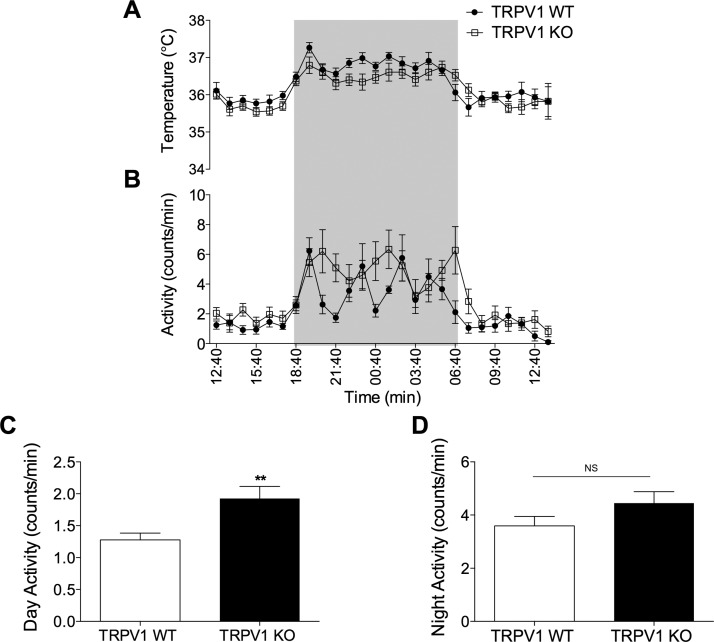
Core body temperature and activity profile of TRPV1 KO mice. Normal circadian rhythm of temperature (*A*) and activity (*B*) of TRPV1 WT mice (closed circles) and TRPV1 KO mice (open squares) over 24 h using radio-telemetry (*n* = 9). Shaded areas in (*A*) and (*B*) denote the dark phase. Average 12-h light-phase activity levels (*C*) and average 12-h dark-phase activity levels (*D*), measured in TRPV1 WT and KO mice (*n* = 9). Results are means ± sem. **P < 0.01 *vs.* WT using 2-tailed Student’s *t* test; NS, not significant.

At baseline conditions, TRPV1 KO mice exhibit a normal body temperature as previously reported (3, 13, 15); we confirmed this by measuring core body temperature under ambient conditions and observed minimal differences in core body temperature results (**Fig. 5*A***). Additionally, TRPV1 KO mice demonstrate hyperkinesis during the light phase (18), a characteristic of thermoregulatory imbalance, which was observed in our results (Fig. 5*B*, *C*). During the dark phase, both groups exhibited equal levels of nocturnal activity (Fig. 5*D*).

### Suppressed sympathetic drive in TRPV1 KO mice

**Figure 6.**
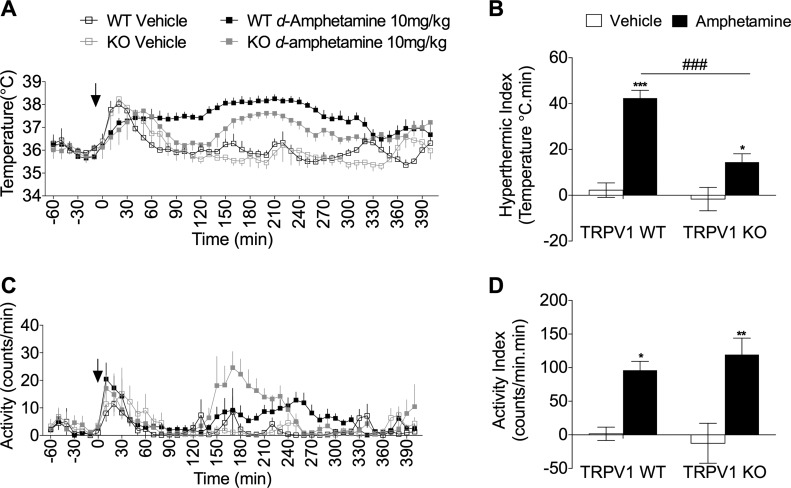
Amphetamine-induced hyperthermia is attenuated in TRPV1 KO mice. *A*) Core body temperature recording following d-amphetamine (10 mg/kg, s.c.; *n* = 7) or vehicle (saline, 5 ml/kg, s.c.; *n* = 5) administration to TRPV1 WT and KO mice. *B*) The hyperthermic index was analyzed as area under the temperature curve during the 120 to 290 min period for vehicle and d-amphetamine-treated mice. *C*) Activity levels following d-amphetamine (10 mg/kg, s.c.; *n* = 7) or vehicle (saline, 5 ml/kg, s.c.; *n* = 5) administration to TRPV1 WT and KO mice. *D*) Respective activity index analyzed as area under the activity curve during the 120 to 290 min period. Results are means ± sem. *P < 0.05, **P < 0.01, and ***P < 0.001 *vs.* control; ^###^*P* < 0.001 *vs.* genotype using 2-tailed Student’s *t* test and 2-way ANOVA and Bonferroni’s *post hoc* test.

It is established that TRPV1 KO mice are immune to the hyperthermia induced by TRPV1 antagonists, and indeed, the data presented in this study confirm the selectivity of TRPV1 antagonist-induced hyperthermia. However, the thermoregulatory response of TRPV1 KO mice to other pharmacologic modulators of core body temperature has not been established. Amphetamines are potent sympathomimetics known to cause substantial hyperthermia in multiple species (38); we utilized d-amphetamine as a positive control and to test the hypothesis that TRPV1 KO mice exhibit a suppressed sympathetic drive. WT mice developed hyperthermia in response to d-amphetamine (10 mg/kg), which peaked at 120 min and was sustained up to 300 min postadministration of d-amphetamine (**Fig. 6*A***). Unlike AMG9810, TRPV1 KO mice exhibited a statistically significant hyperthermia compared to respective control animals (Fig. 6*A*, *B*). However, the hyperthermia was substantially attenuated in comparison with WT mice administered with d-amphetamine, as evident in the hyperthermic index (Fig. 6*B*). Additionally, d-amphetamine caused a significant increase in activity in both TRPV1 WT and KO mice (Fig. 6*C*, *D*), suggesting that the difference in body temperature cannot solely be accounted for by changes in activity levels. Furthermore, it is established that the effects of d-amphetamine on body temperature and activity are independent (39, 40).

**Figure 7.**
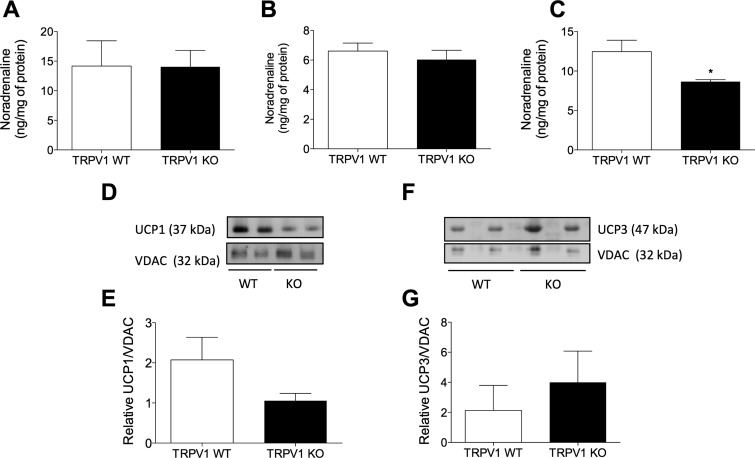
Suppressed sympathetic activity in TRPV1 KO mice. NA concentrations measured in brain (*A*), BAT (*B*), and skin (*C*) samples of TRPV1 WT and KO mice (*n* = 7–8). *D*) Immunoblot of UCP1 and loading control VDAC in BAT mitochondrial protein of TRPV1 WT and KO mice. *E*) Densitometric analysis of the relative expression of UCP1/VDAC (*n* = 5–6). *F*) Immunoblot of UCP3 and loading control VDAC in skeletal muscle mitochondrial protein of TRPV1 WT and KO mice. *G*) Densitometric analysis of the relative expression of UCP3/VDAC (*n* = 5–6). Results are means ± sem. *P < 0.05 *vs.* WT using 2-tailed Student’s *t* test.

To investigate sympathetic activity in TRPV1 KO mice, NA concentrations were measured in naive mice. NA concentrations were unchanged in brain samples from TRPV1 WT and KO mice (**Fig. 7*A***). Furthermore, no distinct differences were determined in NA concentrations in BAT (Fig. 7*B*). Additionally, plasma NA concentrations were not significantly altered (TRPV1 WT, 12.03 ± 1.91 ng/ml *vs.* TRPV1 KO, 26.91 ± 12.93 ng/ml; *n* = 4). However, NA concentrations were significantly reduced in skin samples from TRPV1 KO mice, in comparison with WT mice (Fig. 7*C*).

To assess the physiologic state of BAT in TRPV1 KO mice, basal UCP1 content was measured. The results show that UCP1 expression was decreased in mitochondrial extracts of BAT samples of TRPV1 KO mice, compared to WT counterparts (Fig. 7*D*, *E*), but this did not reach significance. Interestingly, similar results were documented in sensory nerve-depleted rats (5), indicating an important role for sensory nerves and TRPV1 channels in maintaining BAT homeostasis. Skeletal muscle-derived thermogenesis was additionally investigated, and no distinct differences in UCP3 levels were determined (Fig. 7*F*, *G*). Intrascapular BAT weight was significantly increased in TRPV1 KO mice (Supplemental Fig. 2*B*), independent of body mass (Supplemental Fig. 2*A*). Thus, BAT morphology was investigated in TRPV1 WT and KO mice, and a whitening phenotype was observed in KO mice (Supplemental Fig. 2*C*), with increased lipid deposition and size. This increased lipidization is known to be associated with reduced β-adrenoceptor signaling (31). Analysis of relative mRNA expression of thermogenic genes in BAT and GM demonstrated a significant down-regulation of UCP3 in BAT samples of TRPV1 KO mice (**Table 3**), whereas UCP1 exhibited a similar trend.

**TABLE 3.** Relative mRNA expression of thermogenic mediators in naive TRPV1 WT and KO mice

Target	Tissue	TRPV1 WT	TRPV1 KO
UCP1	BAT	1.02 ± 0.08	0.65 ± 0.14
	GM	1.02 ± 0.10	1.23 ± 0.51
UCP2	BAT	1.04 ± 0.19	1.14 ± 0.31
	GM	1.08 ± 0.26	1.01 ± 037
UCP3	BAT	1.01 ± 0.08	0.60 ± 0.03***
	GM	1.12 ± 0.21	1.40 ± 0.46
PGC1-α	BAT	1.01 ± 0.07	1.06 ± 0.33
	GM	1.06 ± 0.25	0.85 ± 0.18
PPAR-γ	BAT	1.18 ± 0.46	0.67 ± 0.28
	GM	1.16 ± 0.37	0.73 ± 0.32
Dio2	BAT	1.02 ± 0.15	0.73 ± 0.31
	GM	1.00 ± 0.05	1.01 ± 0.46

Results are means ± sem. *n* = 6, relative to TRPV1 WT mice. ***P < 0.001 TRPV1 KO *vs.* TRPV1 WT as determined by 2-tailed Student’s *t* test.

## DISCUSSION

Basal body temperature is regulated by TRPV1 in a multitude of species from mice to humans (4). Acute inhibition of TRPV1 results in a profound hyperthermia, associated with increased thermogenesis and skin vasoconstriction. Therein, it would suggest that TRPV1 either tonically promotes heat loss or suppresses heat gain mechanisms. Chronic pharmacologic inhibition of TRPV1 leads to an attenuation of hyperthermia (14), which corresponds well with the normal body temperature observed in TRPV1 KO mice. However, the compensatory mechanism is currently unknown. Moreover, an understanding of the compensatory mechanism may provide insight into the mechanism underlying the hyperthermic response to acute loss of TRPV1 activity, leading to an increased understanding of the basal body temperature regulation.

The initial aim of this study was to confirm that the TRPV1 antagonist, AMG9810, increases body temperature in mice and to verify that the effects are solely mediated by TRPV1. To this end, we showed that AMG9810 (50 mg/kg i.p.) caused a significant increase in the body temperature of WT mice, in accordance with previous studies using a range of TRPV1 antagonists from various chemical classes. TRPV1 KO mice did not develop AMG9810-induced hyperthermia, confirming that no off-target actions were contributing to the effect on thermoregulation. In addition to hyperthermia, analysis of activity levels in WT mice showed a sustained increase in spontaneous activity in AMG9810-treated mice. This increase in activity (hyperkinesis) was previously shown by previous authors, using the antagonist, AMG0347 (18). No increase in activity was observed in KO mice in either our study or that of previous studies (17), and the effect is not stress induced because the study by Garami *et al.* (18) used preimplanted catheters to allow for drug injection without disturbance of the mice (18). Subsequent experiments in the present study showed that mice need to be conscious for hyperthermia to occur because AMG9810 caused no change in body temperature in anesthetized mice. This supports the concept that spontaneous activity may contribute to the role of TRPV1 in thermoregulation.

Once the effect of TRPV1 inhibition on body temperature had been ratified, we aimed to investigate the contribution of proinflammatory cytokines in this hyperthermia. The involvement of cytokines in fever is well known, but their role in hyperthermia is less clear. Sympathomimetics such as MDMA have been shown to possess immunosuppressant effects (41), whereas whole-body heating did not result in an increase in cytokines (42). Therefore, our results establish a lack of involvement of cytokines in TRPV1 antagonist-induced hyperthermia.

Plasma cytokine concentrations were not affected by AMG9810 in the present study, confirming that the increase in body temperature was indeed hyperthermia.

### Stimulation of noradrenergic signaling in the periphery downstream of TRPV1 antagonism

**Figure 8.**
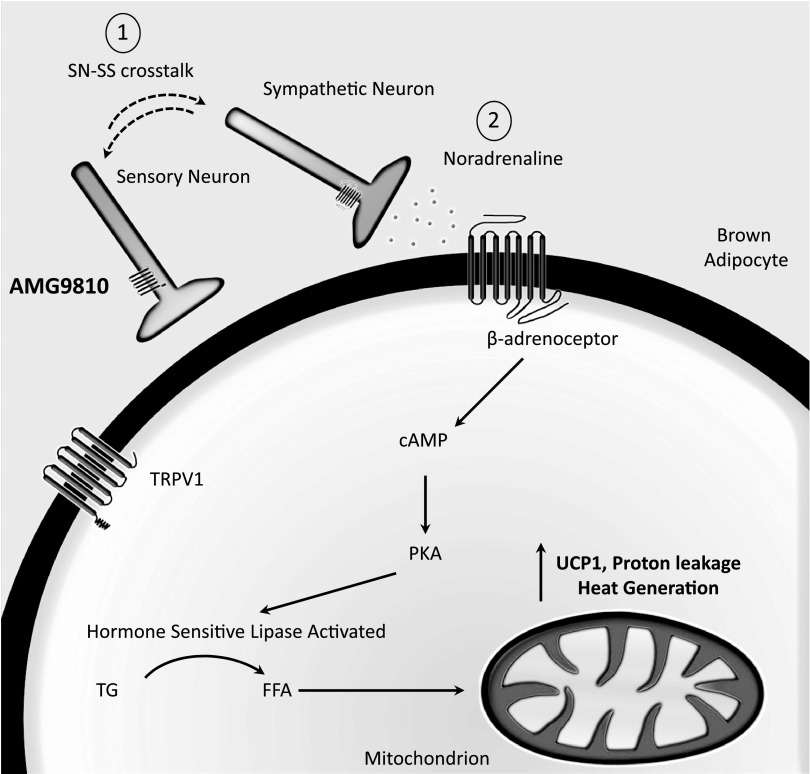
TRPV1 antagonist-induced hyperthermia involves BAT-mediated thermogenesis. Proposed schematic illustrates acute BAT activation following TRPV1 antagonist-induced hyperthermia. Sensory nerves and sympathetic nerves innervate BAT, suggested to mediate feedback loops serving a proposed pathway for crosstalk. Brown adipocytes have been shown to express TRP channels, including TRPV1 (58). AMG9810 transiently disrupts this feedback loop (1), causing hyperthermia mediated *via* an increase in sympathetic activity and NA release (2). NA stimulates β-adrenoceptors and initiates downstream signaling resulting in heat generation. SN-SS, sensory nerve-sympathetic system; TG, triglyceride; FFA, free fatty acid.

Because it was established that acute loss of TRPV1 activity induces a pharmacological hyperthermia, as opposed to cytokine-induced fever, we hypothesized that TRPV1 may be tonically suppressing heat gain mechanisms. More specifically, we hypothesized that TRPV1 tonically suppresses the SNS. Increased activity of the SNS initiates both cutaneous vasoconstriction and BAT thermogenesis and is essential for overall thermoregulation in mammals (7, 8). Thus, we investigated concentrations of the principal sympathetic neurotransmitter, NA, in sympathetic and thermoregulatory organs. NA concentrations were measured in WT mice at the peak hyperthermic period (*i.e.*, 1 h post-AMG9810 or vehicle). AMG9810 did not increase the NA of the brain, although this was not surprising considering that peripheral restriction of TRPV1 antagonists in previous studies did not prevent hyperthermia (43). In contrast, AMG9810 significantly increased the NA content of BAT, suggesting an increase in sympathetic activity and subsequent BAT-mediated thermogenesis (**Fig. 8**). Interestingly, a significant decrease in NA content in skin samples of AMG9810-treated animals was detected. NA concentrations in the peripheral cutaneous vasculature are tightly regulated, with acute metabolism/uptake in order to preserve vascular tone (44). Thus, it is possible that this reduction was facilitated by enhanced metabolism/uptake to preserve vascular tone. Increased plasma concentrations of NA in AMG9810-treated mice indicated a possibility of increased NA outflow to the periphery, which has been previously observed in cold-induced thermogenesis in rodents (45).

Having determined an increase in NA concentrations in BAT and plasma following acute inhibition of TRPV1 with AMG9810, we sought to confirm the role of the SNS in the thermoregulatory effect of TRPV1 by adrenoceptor inhibition. In particular, we aimed to selectively inhibit various adrenoceptor subtypes to determine the precise adrenoceptor responsible for the hyperthermic response to AMG9810. Pretreatment with the selective α_1_-adrenoceptor antagonist decreased both the magnitude of AMG9810-induced hyperthermia and the hyperthermia index. These results are in agreement with the role of α_1_-adrenoceptors in mediating cutaneous vasoconstriction downstream of NA (37), and thus, treatment with prazosin may have facilitated enhanced heat loss in these mice. Furthermore, prazosin has previously been shown to potently inhibit MDMA-mediated hyperthermia in rodents (26). However, we were unable to eliminate the possibility that the cutaneous vasculature of our mice was maximally vasoconstricted prior to injection of AMG9810 because the room temperature (22°C) was subneutral for mice. In this case, we propose that although naive mice would be maximally vasoconstricted at such a subneutral temperature, α_1_-adrenoceptor receptor activation would not usually be maintained in hyperthermic mice to facilitate heat loss. In the case of TRPV1 antagonist-induced hyperthermia, we propose that this α_1_ receptor activation is maintained, preventing heat loss.

In contrast to prazosin, both the nonselective β-adrenoceptor antagonist, propranolol, and mixed α-/β-adrenoceptor antagonist, labetalol (36), resulted in a marked delay in the onset of the AMG9810-induced hyperthermia and a significant decrease in the hyperthermic index. Conversely, selective blockade of β_1_-adrenoceptors with metoprolol resulted in a potentiation of AMG9810-induced hyperthermia, whereas selective inhibition of other adrenoceptor subtypes had no effect on the AMG9810-induced hyperthermic index. In contrast to this study, metoprolol has been previously shown to decrease insulin-mediated thermogenesis (46). However, similar to TRPV1 antagonists, metoprolol decreases baseline forearm blood flow (46) and, therefore, has the potential to exacerbate the hyperthermia in this case. It was particularly interesting that atypical β_3_-adrenoceptor was not involved in AMG9810-induced hyperthermia because it has previously been shown to be involved in 3,4-MDMA-induced hyperthermia (26) and is considered to play a role in lipolysis and thermogenesis (47). This may be due to compensatory effects between adrenoceptors because mice with targeted deletion of β_1_-, β_2_-, or β_3_-adrenoceptors exhibit normal thermogenesis following a cold challenge (48), whereas the triple KO is cold intolerant (49). Overall, the series of experiments from the present study showed that acute inhibition of TRPV1 causes hyperthermia *via* a combination of β-adrenoceptor subtypes and supported the hypothesis that TRPV1 tonically suppresses sympathetic activity (*i.e.*, TRPV1 acts upstream of the SNS to modulate body temperature).

To further confirm the interaction between TRPV1 and the SNS in thermoregulation, we investigated the expression of various proteins, involved in thermogenesis, that are under sympathetic control in BAT and skeletal muscle. UCP1 is the most potent thermogenic protein in mammals, but neither its mRNA nor protein levels were significantly altered in BAT from AMG9810-treated mice. These results suggest that if UCP1 is involved in the hyperthermia induced by acute TRPV1 inhibition, then UCP1 activity is probably altered, as opposed to UCP1 expression, at least during the first hour after AMG9810 administration. Protein levels of the skeletal muscle-abundant homolog, UCP3, were increased in GM muscle of the same animals, which is interesting considering the pivotal role that UCP3 plays in amphetamine-mediated thermogenesis (33).

Despite a lack of change in UCP1 mRNA and protein levels, a significant induction in the mRNA expression of downstream signaling mediators such as PGC1-α was determined in BAT samples from AMG9810-treated mice. It is known that the expression of UCP1 is highly fluidic and is driven by several transcriptional components, including PGC1-α (34). PGC1-α is known to regulate oxidative metabolism by increasing mitochondrial gene expression and function (35). PPAR-γ, a regulator of UCP1 expression (34), was modestly increased by AM9810. In contrast, a significant induction of Dio2 was observed, implicating the HPT axis in AMG9810-induced hyperthermia (50). Dio2 has been shown to be an essential component in the thyroid-sympathetic synergism in BAT-mediated thermogenesis (51); a significant induction in Dio2 has been shown during cold exposure, which facilitates the accelerated conversion of the biologically inactive thyroxine (T4) to 3,3′,5-triiodothyronine (T3) (52, 53), which increases adrenergic responsiveness in a positive feedback mechanism (53). Additionally, Dio2 can directly act as a transcriptional factor modulating UCP1 biogenesis (51). Taken together, our results suggest that acutely, AMG9810 initiates a thermogenic response in BAT, which is indicative of UCP1 activation.

### TRPV1 KO mice exhibit suppressed peripheral sympathetic activity

We subsequently sought to explore the thermoregulatory profile of TRPV1 KO mice. The TRPV1 KO thermoregulatory phenotype has been explored before where basal body temperature did not significantly differ from WT mice, suggesting that a compensatory adjustment has been made to regulate body temperature. As previously reported, we observed an increase in locomotor activity in KO mice during the light phase of their light-dark cycle (18). We also observed this hyperkinesis in TRPV1 KO mice, whereas both groups exhibited equal levels of nocturnal, dark-phase activity. The hyperactivity has been suggested as a compensatory mechanism for the altered thermoregulatory profile these mice display when thermally challenged (18). In addition to activity changes, we hypothesized that SNS activity is suppressed in TRPV1 KO mice.

To test sympathetic dysfunction in TRPV1 KO mice, the sympathomimetic drug, d-amphetamine, was utilized to assess the temperature responses in these mice. TRPV1 KO mice demonstrated a significantly attenuated hyperthermia during the period in which hyperthermia was significant in WT animals. Amphetamine administration has previously been shown to increase both BAT and rectal temperature in a manner that can be inhibited with propranolol (54), similar to the findings with AMG9810. In the present study, d-amphetamine caused a significant increase in activity in both TRPV1 WT and KO mice, indicating that any difference in body temperature between the 2 genotypes cannot solely be accounted for by changes in activity levels. In addition, it may suggest that the effect of d-amphetamine on body temperature is sympathetically mediated, whereas the effect on activity is not. Indeed, it is well known that amphetamine induces dopamine release in the *nucleus accumbens* of the brain, a region associated with dopamine-induced locomotor activity. Furthermore, it was reported that amphetamine-induced hyperactivity and dopamine release are enhanced in mice lacking the M(5) receptor, suggesting a role for muscarinic receptors in the process (55). Overall, the results in this component of the present study showed a reduced capacity of TRPV1 KO animals to respond to a sympathomimetic drug, suggesting that sympathetic drive has been reduced as a compensatory mechanism to normalize body temperature in these animals.

As sympathetic activity in TRPV1 KO animals appeared to be reduced, we carried out experiments to test whether NA levels in brain, skin, and BAT from KO animals were lower than those of their WT counterparts. The NA concentration measured in brain and BAT samples did not significantly differ between WT and KO animals, but NA concentrations in skin samples of TRPV1 KO mice were significantly decreased compared to WT controls, suggesting a compensatory decrease in NA production or metabolism. As BAT NA content was not different between the 2 groups, we sought to assess the thermogenic capacity of BAT by measurement of UCP1 content. The results illustrated a trend toward decreased UCP1 expression in mitochondrial extracts of BAT samples of TRPV1 KO mice, compared to WT controls. Interestingly, similar results were documented in sensory nerve-depleted rats (5), indicating an important role for sensory nerves and TRPV1 channels in maintaining BAT homeostasis. Intrascapular BAT weight was significantly increased in TRPV1 KO mice along with morphologic changes (*i.e.*, a whitening phenotype in KOs), with increased lipid deposition and size. This increased lipidization is known to be associated with reduced β-adrenoceptor signaling, associated with reduced sympathetic activity (31).

In conclusion, this study confirms previous findings that TRPV1 plays a pivotal role in the regulation of body temperature. New evidence from the present study demonstrates that TRPV1 is acting upstream of the SNS to exert this effect. Acute removal of this regulatory activity *via* administration of antagonists results in an increased sympathetic drive and manifestation of hyperthermia. Furthermore, genetic deletion of TRPV1 results in a compensatory suppression of sympathetic activity and/or associated thermoregulatory pathways. The fundamental role of the SNS in thermoregulation has been known for many years, but this is the first study to show that this regulatory effect is controlled by TRPV1 at a basal level, providing a potential therapeutic target for hypersympathetic thermoregulatory disorders.

## Supplementary Material

Supplemental Data

## Abbreviations

AUC: area under the curve
BAT: brown adipose tissue
Dio2: type II iodothyronine deiodinase
DSI: Data Science International
GM: *gastrocnemius*
HPRT: hypoxanthine phosphoribosyltransferase
HPT: hypothalamic-pituitary-thyroid
HRP: horseradish peroxidase
KO: knockout
MDMA: methylenedioxy methamphetamine
NA: noradrenaline
PGC1-α: proliferator-activated receptor γ coactivator 1-α
pNPP: p-nitrophenyl phosphate
PPAR-γ: peroxisome proliferator-activated receptor γ
qPCR: quantitative PCR
SNS: sympathetic nervous system
TRPV1: transient receptor potential vanilloid 1
UCP: uncoupling protein
VDAC: voltage-dependent anion-selective channel
WT: wild-type
