# Supplementary material for: The sympathetic nervous system is controlled by transient receptor potential vanilloid 1 in the regulation of body temperature
Source: FASEB J. 2015 Jul 1;29(10):4285–98. doi: 10.1096/fj.15-272526 (PMC4650996; doi:10.1096/fj.15-272526)
Supplement: Supplemental Data [file supp_29_10_4285__index.html]

The sympathetic nervous system is controlled by transient receptor potential vanilloid 1 in the regulation of body temperature — The sympathetic nervous system is controlled by transient receptor potential vanilloid 1 in the regulation of body temperature — Supplemental Data 

# The sympathetic nervous system is controlled by transient receptor potential vanilloid 1 in the regulation of body temperature

## Supplemental Data

- Supplemental Data
- Supplemental Data
