# Supplementary material for: The sympathetic nervous system is controlled by transient receptor potential vanilloid 1 in the regulation of body temperature
Source: FASEB J. 2015 Jul 1;29(10):4285–98. doi: 10.1096/fj.15-272526 (PMC4650996; doi:10.1096/fj.15-272526)
Supplement: Supplemental Data [file supp_fj.15-272526_Supplemental_Figure1.pdf]

Supplemental Figure 1.

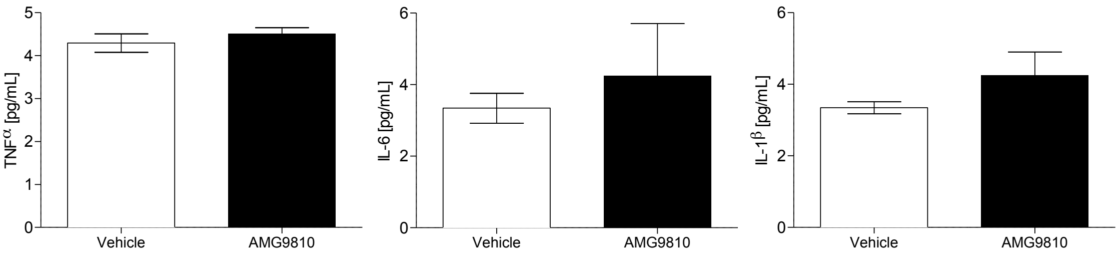

Supplemental Figure 1.

Plasma cytokine concentrations are unchanged by AMG9810-mediated hyperthermia. TNFα, IL6, IL1β concentrations were investigated in plasma from AMG9810 or vehicle-treated WT mice. Results are mean + s.e.m. n=5-6.
