# Supplementary material for: The sympathetic nervous system is controlled by transient receptor potential vanilloid 1 in the regulation of body temperature
Source: FASEB J. 2015 Jul 1;29(10):4285–98. doi: 10.1096/fj.15-272526 (PMC4650996; doi:10.1096/fj.15-272526)
Supplement: Supplemental Data [file supp_fj.15-272526_Supplemental_Figure2.pdf]

Supplemental Figure 2.

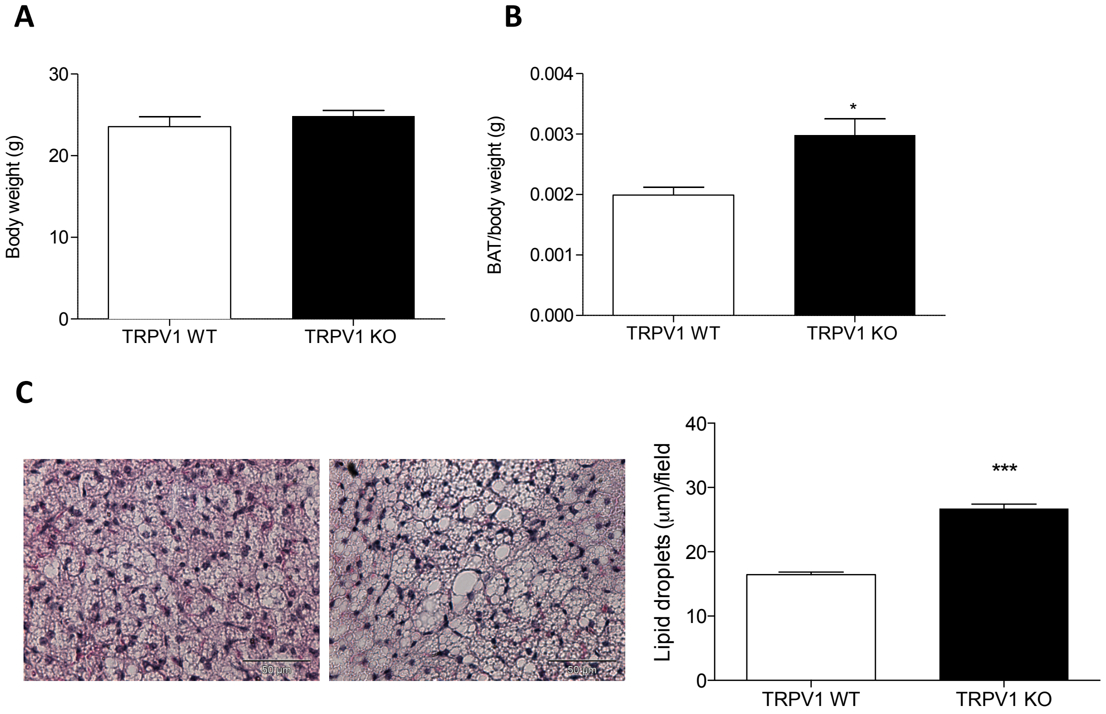

Supplemental Figure 2

Increased lipisation in TRPV1 KO brown adipose tissue.

(A) Body mass of juvenile TRPV1 WT and KO mice (n=4-6). (B) Intrascapular BAT mass normalised to body weight is increased in TRPV1 KO mice (n=4-6). (C) H&E staining of BAT from naive TRPV1 WT (left) and KO (right) panel, acquired at x40 magnification; scale bar: 50 μm. Quantification of lipid droplets diameter (n=4-6). *p<0.05, ***p<0.01 vs WT using two-tailed Student's *t* test.
